# MB-AI-His: Histopathological Diagnosis of Pediatric Medulloblastoma and its Subtypes via AI

**DOI:** 10.3390/diagnostics11020359

**Published:** 2021-02-20

**Authors:** Omneya Attallah

**Affiliations:** Department of Electronics and Communications Engineering, College of Engineering and Technology, Arab Academy for Science, Technology and Maritime Transport, Alexandria 1029, Egypt; o.attallah@aast.edu

**Keywords:** pediatric medulloblastoma (MB) diagnosis, histopathology, computer-aided diagnosis (CADx), convolutional neural network (CNN), discrete wavelet transform (DWT), discrete cosine transform (DCT), principal component analysis (PCA)

## Abstract

Medulloblastoma (MB) is a dangerous malignant pediatric brain tumor that could lead to death. It is considered the most common pediatric cancerous brain tumor. Precise and timely diagnosis of pediatric MB and its four subtypes (defined by the World Health Organization (WHO)) is essential to decide the appropriate follow-up plan and suitable treatments to prevent its progression and reduce mortality rates. Histopathology is the gold standard modality for the diagnosis of MB and its subtypes, but manual diagnosis via a pathologist is very complicated, needs excessive time, and is subjective to the pathologists’ expertise and skills, which may lead to variability in the diagnosis or misdiagnosis. The main purpose of the paper is to propose a time-efficient and reliable computer-aided diagnosis (CADx), namely MB-AI-His, for the automatic diagnosis of pediatric MB and its subtypes from histopathological images. The main challenge in this work is the lack of datasets available for the diagnosis of pediatric MB and its four subtypes and the limited related work. Related studies are based on either textural analysis or deep learning (DL) feature extraction methods. These studies used individual features to perform the classification task. However, MB-AI-His combines the benefits of DL techniques and textural analysis feature extraction methods through a cascaded manner. First, it uses three DL convolutional neural networks (CNNs), including DenseNet-201, MobileNet, and ResNet-50 CNNs to extract spatial DL features. Next, it extracts time-frequency features from the spatial DL features based on the discrete wavelet transform (DWT), which is a textural analysis method. Finally, MB-AI-His fuses the three spatial-time-frequency features generated from the three CNNs and DWT using the discrete cosine transform (DCT) and principal component analysis (PCA) to produce a time-efficient CADx system. MB-AI-His merges the privileges of different CNN architectures. MB-AI-His has a binary classification level for classifying among normal and abnormal MB images, and a multi-classification level to classify among the four subtypes of MB. The results of MB-AI-His show that it is accurate and reliable for both the binary and multi-class classification levels. It is also a time-efficient system as both the PCA and DCT methods have efficiently reduced the training execution time. The performance of MB-AI-His is compared with related CADx systems, and the comparison verified the powerfulness of MB-AI-His and its outperforming results. Therefore, it can support pathologists in the accurate and reliable diagnosis of MB and its subtypes from histopathological images. It can also reduce the time and cost of the diagnosis procedure which will correspondingly lead to lower death rates.

## 1. Introduction

Brain tumors are very common pediatric solid tumors accounting for around 25% of all types of pediatric cancers [1]. Among children below 15 years old, the brain tumor is the second major reason for mortality after severe lymphoblastic leukemia [2]. It is stated that more than 1500 kids in America and 1859 kids in Britain were diagnosed annually with cancer during 2014 to 2016; 15% of them consequently died [3]. About 55–70% are pediatric brain tumors. Including them, around 15% of brain tumors are medulloblastoma (MB). MB is the foremost common pediatric malignant brain tumor [4]. MB is also the main reason for cancer-related illness and death among children [5,6]. It develops inside the cerebellum on the posterior part of the brain and rapidly grows. Because MB is a pediatric brain tumor, there is a necessity to attain a serious examination, so as not to result in an over or under treatment, which in both cases leads to an excessive death rate [7]. It has several subtypes. As stated in [8], the accurate diagnosis of pediatric subtype has enhanced the 2 and 5 year survival rates. Late diagnosis of MB and its subtypes may cause acute side effects. This is because the cerebellum controls all body motion and synchronization. Therefore, the accurate diagnosis of pediatric MB and its subtypes is essential to reduce mortality rates and select the appropriate treatment plan that prevents its progression.

Magnetic Resonance Imaging (MRI) is the common scanning modality utilized to scan and diagnose children’s brain tumors [9]. However, there are some difficulties that face radiologists to diagnose pediatric MB subtypes using MRI [1]. This is because various brain tumor categories do not constantly reveal obvious variations in the visible manifestation of the MRI scan [10]. Moreover, employing just traditional MRI to deliver a diagnosis could possibly lead to an inaccurate decision [11]. Therefore, another imaging modality is preferred to diagnose MB and its subtypes [1]. Currently, the classification of pediatric MB and its subtypes is accomplished by a histopathological imaging, which is the gold standard to attain accurate diagnosis of MB and its subtypes [11,12]. The treatment process relies on the MB subtype classification, as the level of aggressiveness varies from subtype to another. Therefore, the correct classification of the MB subtype is of great importance [13]. However, limited works have studied the classification of MB subtypes using image processing and machine learning techniques [12]. Diagnosing MB is useful to define the destructive MB subtypes that need severe and quick treatments [14]. There are four subtypes of MB that depend on the histological visual appearance according to the World Health Organization (WHO) classification [15]. These subtypes compromise the classic, desmoplastic MB with extensive nodularity and large cell/anaplastic MB. Discriminating among these subtypes of MB is hard mostly because of the complication among the patterns observed in the histopathology scans as well as the cell shape, association, size, and the alignment inconsistency for the distinct malignant classes of the tumor [16]. Conventional methods for the diagnosis of MB and its subtype depend on the recognition of useful, significant, and discriminating features of the visible structural patterns located in the histopathological images. However, unfortunately, these methods generally do not succeed at observing the mixture of complicated patterns that exist in the histopathological images which are very similar for the four MB subtypes that makes the classification process a challenging task [17]. Moreover, the pathological analysis is complex, time-consuming, and is subjective to the pathologist’s knowledge and experience [18]. Professional pathologists might deliver different decisions regarding the MB subtype [19,20]. Additionally, the limited availability of pathologists is a serious hurdle in the analysis of histopathological images. This deficiency occurs mostly in the developed and developing countries. The lack of pathologists rises the burden on the present pathologists [21]. This emphasizes the necessity for powerful automatic approaches or mixtures of approaches to overcome the challenging tasks that appear during the manual analysis of histopathological images. Such automatic techniques will be able to lower the load made by the pathologist in classifying MB and its subtypes, and further support them in achieving precise MB diagnosis [18]. 

In the last decade, there have been huge advancements in artificial intelligence (AI) methods comprising machine learning (ML) and deep learning (DL) approaches. Computer-aided diagnosis (CADx) schemes based on ML and DL approaches have led to significant enhancements in the automatic diagnosis of pediatric MB and its subtypes. CADx could assist pathologists in the automatic analysis of histopathological images, thus decreasing the cost of diagnosis [22]. Several CADx systems have been proposed to solve related medical problems [23,24,25,26,27]. However, less work has been made to classify the childhood MB and its subtypes from histopathological images using ML and DL techniques, due to the lack of data availability. The main aim of this paper is to propose a reliable and time-efficient system called MB-AI-His for the automatic diagnosis of pediatric MB and its subtypes from histopathological images. MB-AI-His is a mixture of deep learning and machine learning methods. It is fully automated to avoid manual diagnosis made by pathologists, help them in achieving an accurate diagnosis, and identify the four subtypes of childhood MB. MB-AI-His overcomes the limitations and drawbacks of the related studies. First, it is a reliable system capable of classifying the four subtypes of childhood MB with high accuracy instead of only one subtype as obtained by several related works. Second, it merges the advantages of both deep learning and textural analysis through a cascaded manner. This is done by initially extracting spatial DL features from three convolutional neural network (CNN) approaches, then using the discrete wavelet transform (DWT) method to further extract textural features from the DL features which form spatial-time-frequency features. Third, it combines the spatial-time-frequency features extracted from the three CNNs after passing through the DWT to benefit from each CNN architecture. Fourth, it fuses the three spatial-time-frequency features using the discrete cosine transform (DCT) and principal component analysis (PCA) methods to reduce the huge dimension of features and the training execution time. Note that one of the main challenges in classifying the subtypes of the pediatric MB is the availability of the dataset. 

The novelty of the paper can be summarized into the following contributions:

- Few related studies were conducted for classifying the four subtypes of pediatric MB. Most of them did not achieve very high performance, so they are not reliable. In this paper, a reliable CADx is constructed, called MB-AI-His, that can classify the four subtypes of pediatric MB with high accuracy.
- Most previous studies depend only on textural analysis-based features or deep learning features that were used individually to perform classification; however, MB-AI-His merges the benefits of the DL and textural analysis feature extraction methods through a cascaded manner.
- The cascaded manner initially uses three deep CNNs to extract spatial features. Then, these spatial features enter a DWT which is a textural analysis-based method that generates time-frequency features ending up by generating spatial-time-frequency features.
- Developing spatial-time-frequency features instead of using only spatial features as accomplished by most of the related studies.
- Almost all the related studies used an individual feature set to construct their classification model; however, MB-AI-His fuses the three spatial-time-frequency features generated from the three CNNs and DWT.
- The fusion is done through DCT and PCA to generate a time-efficient CADx system and lower the feature space dimension as well as the classification training time which was one of the limitations in the previous related work.

The paper is organized as follows. Section 2 describes the related studies with their limitations. Section 3 introduces the dataset used as well as the DL and ML approaches and the proposed MB-AI-His. Section 4 presents the parameters’ settings and the performance metrics used to evaluate the results of MB-AI-His. The results of the proposed MB-AI-His are shown in Section 5. Section 6 discusses the main results of MB-AI-His, and finally, Section 7 concludes the paper.

## 2. Related Work

This section illustrates the methods and results achieved using related studies. The related studies based on histopathological images along with their limitation are shown in Table 1. The techniques [12,16,28,29,30] stated in Table 1 suffer from several limitations. First, most of them are based only on handcrafted feature extraction approaches which have some number of parameters that should be manually adjusted, which involves additional time for training the classification model. Moreover, some of them depend on only textural-based feature extractors which might not succeed to explain the feature patterns existing in the training instances that the data-driven method is capable to find [28]. Additionally, they used only individual types of features to construct their models. They were also all based on a very small dataset containing only 10 images. Finally, they were all constructed to distinguish between only anaplastic and non-anaplastic pediatric MB, which is only one subclass of childhood MB (binary classification problem). The drawbacks of the methods in [7] and [31] are using conventional handcrafted features based on either textural analysis, color, or morphological operations to train the support vector machine SVM classifier to classify the four subtypes of MB. Moreover, the CADx proposed in [13] studied the fusion of only textural features to train their model and perform the classification task. The authors in [32] used only DL features to train an SVM classifier to classify the four classes of childhood MB. They used only two types of DL methods individually for the classification task, each of them is of huge dimension. They did not combine several DL features extracted from several CNNs to benefit from each CNN architecture. The authors in [32] only used two pre-trained CNNs individually. Moreover, the classification time executed using these CNNs is high. Besides, none of the above methods combined DL features with textural features. Finally, most of them did not achieve a very high accuracy, which means they are not reliable.

## 3. Materials and Methods

### 3.1. Childhood MB Dataset Description

**Figure 1:**
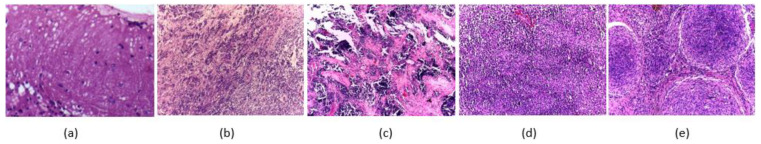
Samples of the childhood pediatric MB images: (**a**) normal, (**b**) classic, (**c**) desmoplastic, (**d**) large cell, (**e**) nodular.

The Guwahati Medical College and Hospital GMCH and Guwahati Neurological Research Centre (GNRC) were both employed as collaborating medical institutes in collecting childhood MB dataset. The dataset used in constructing MB-AI-His was collected from only patients experiencing childhood MB. All these patients are of age lower than 15 years. Few blocks of the data were generated from children under 15 years of age who were identified with childhood MB at the neurosurgery department of GMCH. The samples were gathered from the tissue blocks and utilized as an element of the post-operative process. Blocks of tissues were then stained using hematoxylin and eosin (HE) at Ayursundra Pvt where pathological assistance was delivered by a local medical specialist. The dataset was collected from 15 children from whom the samples were gathered. Afterward, the slide’s scans and the region of interest were observed for ground truth by a qualified pathologist at the Pathological Department of GNRC. Next, pictures of the region of interest where microscopic images were taken at magnification 10x were saved in JPEG format. These images were captured using a Leica 1CC50 HD microscope. The dataset contains images for the four subtypes of MB tumors. The total number of images is 204. The number of images for the classic, desmoplastic, large cell, and nodule MB subtypes is 59, 42, 30, and 23, respectively. Whereas the number of normal images that do not contain signs of MB is 50. Details of the dataset can be found in [33]. The dataset can be found at [34]. Samples of normal and MB subtypes’ images available in the dataset are shown in Figure 1 which are (a) normal, (b) classic, (c) desmoplastic, (d) large cell, and (e) nodular.

### 3.2. Deep Learning Approaches

Deep learning (DL) approaches are a new branch of machine learning techniques that arose as a solution to overcome the limitations of the traditional artificial neural network (ANN) when analyzing images. The traditional ANN does not take into account the benefit of the underlying spatial information located in images [35,36,37]. There are several architectures for DL. Among them is the convolutional neural network (CNN), which is the most used architecture for medical problems, especially dealing with medical images [38,39,40]. A CNN contains a huge number of layers; thus, it is denoted deep networks. It consists of convolutional layers, non-linear activation layers, pooling layers, and fully connected (FC) layers. Instead of supplying the whole image to every neuron, the convolutional layer of the CNN convolves a region of the image (equivalent to the size of the filter) with a filter of compact size. This filter passes through the whole regions of the image in the previous layer, one region (equivalent to the size of the filter) at a time. The output of the filter utilized in the previous layer is known as a feature map. Every location leads to the activation of the neuron and the outputs are stored in the feature map [41]. Three state-of-the-art CNN architectures are used in this paper including ResNet-50, DenseNet-201, and MobileNet CNNs.

#### 3.2.1. ResNet-50

**Figure 2:**
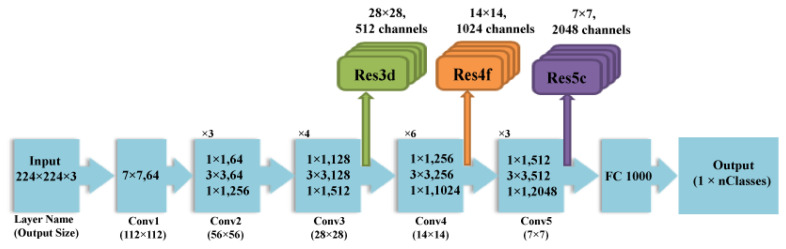
The structural design of ResNet 50 CNN.

The ResNet is considered to be one of the powerful and latest CNNs. It achieved the first position in the ImageNet Large Scale Visual Recognition Challenge ILSVRC and Common Objects in Context COCO 2015 competition [42]. ResNet can efficiently converge with acceptable computation cost even with increasing the number of layers, which is not the case with AlextNet and Inception CNNs [40,43]. This is because He et al. [42] delivered a new structure that depends on deep residual learning. This structure includes cutoffs (called residuals) inside the layers of a traditional CNN to cross over some convolution layers at a time. Such residuals boost the performance of the CNN. Moreover, these residuals accelerate and smoothen the convergence procedure of the CNN despite the huge amount of deep convolution layers [26]. ResNet-50 CNN is employed in the paper which is 50 layers deep. The architecture of ResNet-50 is shown in Figure 2. The dimensions of the various layers of ResNet 50 CNN are shown in Table 2.

#### 3.2.2. DenseNet-201

**Figure 3:**
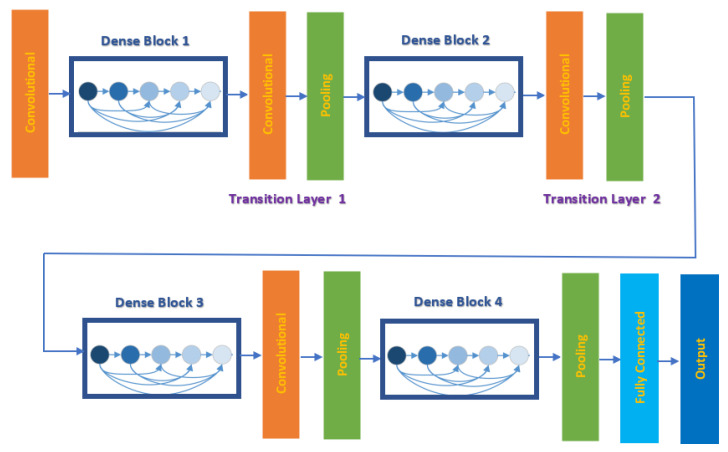
The architecture of DenseNet-201 CNN.

Recent studies have shown that deep CNNs could be substantially deeper, more precise, and have efficient training ability if constructed with smaller links among layers close to input and output. For this reason, Huang et al. [44] in 2017 introduced a new CNN architecture based on the previous short connections called Dense Convolutional Network (DenseNet). This network joins every single layer to all other layers in a feed-forward process. Whereas traditional CNN with Z layers have Z links, one within each layer and its subsequent layer, DensNet consists of Z(Z+1)/2 successive links. For every single layer, the feature maps of the whole preceding layers are employed as inputs, whereas its feature maps are employed as inputs into the entire succeeding layers. This network benefits from its great capability to decrease the vanishing-gradient problem, strengthen feature distribution, enhance feature recovers, and significantly lower the number of parameters. DenseNet-201 is employed in this study, which is 201 layers deep. The architecture of DenseNet-201 is shown in Figure 3. The dimensions of the various layers of DenseNet-201 CNN are displayed in Table 3.

#### 3.2.3. MobileNet

**Figure 4:**
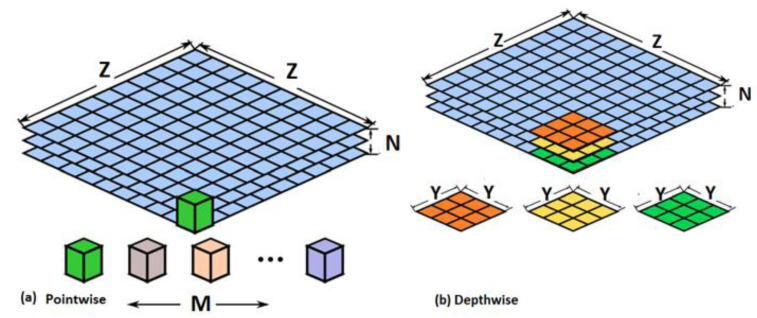
The architecture of MobileNet CNN: (**a**) pointwise convolution layer, (**b**) depthwise (dw) convolution layer.

To benefit from the powerful capability of CNN while making it more usable, practical, and time-efficient, a lightweight CNN called MobileNet was proposed [45]. It was created to enhance the instantaneous performance of CNN under hardware restrictions. MobileNet is capable of lowering the amount of parameters devoid of surrendering accuracy. It only requires 1/33 of the parameters needed for VGG-16 CNN to attain similar accuracy using 1000 images of ImageNet. It consists of point-wise layers (pw) and depth-wise layers (dw). The latter are convolutional layers of size 3 × 3 kernels, whereas the former are convolutional layers of size 1 × 1 kernels. These layers are handled using the activation function rectified linear unit and the batch normalization algorithm [46]. It contains 19 deep layers. Figure 4 shows the structure of the pointwise and depthwise convolution layers, where Z × Z is the size of the feature map, N is the input channel, M is the output channel, and Y × Y is the kernel size for the depthwise convolution layer. Table 4 shows the structure of MobileNet CNN. 

### 3.3. Proposed MB-AI-His 

**Figure 5:**
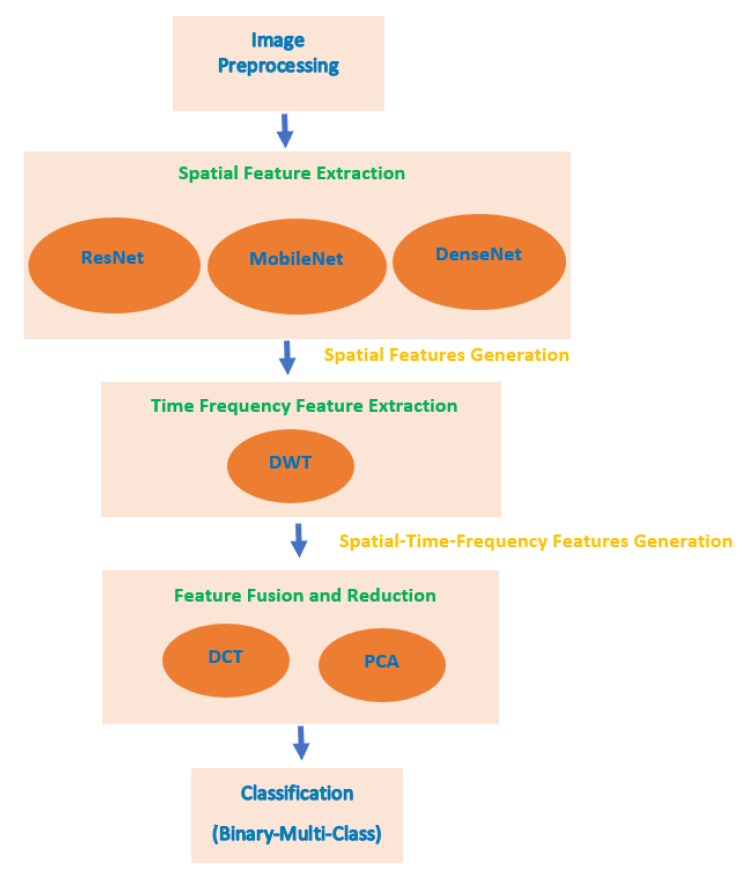
A block diagram of the proposed MB-AI-His.

MB-AI-His perform the automatic diagnosis of pediatric MB and its subtypes from the histopathological images in two levels. The first level classifies the images into normal and abnormal (binary classification level), the second level classifies the abnormal images containing MB tumor into the four subtypes of childhood MB tumor (multi-classification level). MB-AI-His consists of five stages which are image preprocessing, spatial feature extraction, time-frequency feature extraction, feature fusion and reduction, and classification stages. In the image preprocessing stage, images are resized and augmented. In the spatial feature extraction stage, spatial features are extracted from three deep learning CNNs. In the time-frequency feature extraction stage, time-frequency features are extracted using the DWT method. In the feature fusion and reduction stage, the feature sets extracted in the previous stage are fused using DCT and PCA feature reduction techniques. Figure 5 shows a block diagram of the proposed MB-AI-His. 

#### 3.3.1. Image Pre-Processing 

In this stage, for the first level of the proposed CADx, 50 images are selected at random from the four subtypes of childhood MB. This step is made to balance the normal and abnormal classes to 50 images for the binary classification task. Next, for both levels of the proposed MB-AI-His, images are resized to 224 × 224 × 3 to fit the size of the input layer of each CNN. Afterward, these images are augmented. This augmentation step is necessary to elevate the number of images of a dataset to prevent the classification model from overfitting [40,47]. The augmentation methods employed in MB-AI-His to generate new microscopic images from the training images are flipping in x and y directions, translation (−30,30), scaling (0.9,1.1), and shearing (0,45) in x and y directions.

#### 3.3.2. Spatial Feature Extraction

Three deep pre-trained CNNs are utilized with transfer learning. Transfer learning is the capacity to attain matches among distinct data or information to facilitate the training progression of another classification task that has similar mutual elements. This means that the pre-trained CNN can understand representations from large data like ImageNet, and then utilize these demonstrations in other areas having the equivalent classification problem [37]. It is commonly used in the medical field, as finding medical datasets of massive size and mostly labeled as ImageNet dataset is a challenge [35,38]. Transfer learning is also done to allow the CNN to be used as a feature extractor. In this stage, after modifying the FC layers of the three CNNs to be equivalent to the number of classes of the childhood MB dataset (2 in case of binary level and 4 for multiclass level) instead of the 1000 class of ImageNet, spatial features are extracted using three deep pre-trained CNNs including ResNet-50, DenseNet-201, and MobileNet CNNs. These features are taken out from the “global average pooling 2D layer” of ResNet-50, DenseNet-201, and MobileNet CNNs. The dimensions of these spatial deep features are 2048, 1280, and 1920 for ResNet-50, MobileNet, and Dense-Net-201 CNNs respectively as shown in Table 5.

#### 3.3.3. Time-Frequency Feature Extraction

In this stage, time-frequency features are extracted using the discrete wavelet transform (DWT) method. The DWT is a textural analysis based-method that is commonly used in the medical field [48,49,50]. It offers time-frequencies description by decomposing data via a set of perpendicular basis functions. The DWT consists of a group of transforms; everyone has a distinct class of wavelet basis functions. To analyze a 1-D data, a 1-D DWT is employed, which convolve low pass and high pass filters with the input data. Next, a dyadic decimation process is executed which is a down-sampling procedure usually made to reduce the aliasing distortion. Once the 1-D DWT is operated to the 1-D input data, two clusters of coefficients are produced which are the approximation coefficients CA_1_, and detail coefficients CD_1_ [48]. This process can be repeated for the approximation coefficients CA_1_ to attain the second level of decomposition, and again, two sets of coefficients will be created; the second level approximation coefficients CA_2_, and detail coefficients CD_2._ This process can be further performed to produce multi-decomposition levels of DWT. In this stage, one level of DWT is performed for each spatial feature extracted from each CNN of the previous stage. Meyer wavelet (dmey) is utilized as a wavelet basis function. CD_1_ corresponds to the detailed coefficients of the first level of DWT. These details coefficients are produced when passing the image through a high pass filter [51]. In medical images, the details of the images that help in the diagnosis are found in the high frequencies [52,53,54]. Therefore, only CD_1_ coefficients are chosen in this step, as they contain most of the information available in the data, and also to reduce the huge dimension of the features extracted in the earlier stage. Finally, spatial-time-frequency feature sets will be generated at this stage having dimensions of 1074, 1010, and 690 coefficients after applying to ResNet-50, Dense-201, and MobileNet spatial DL features. This step is made to benefit from the advantages of both the DL and DWT textural analysis feature extraction methods. It is also done to verify that the spatial-time-frequency representations are better than the spatial representations.

#### 3.3.4. Feature Fusion and Reduction

To merge the privilege of each of the deep learning techniques used as feature extractors with textural analysis-based features, a fusion process is made in this stage using DCT and PCA. These methods are also used to lower the huge dimension of features. The numbers of DCT coefficients and principal components are chosen using a sequential forward search strategy.

- **DCT** is regularly applied to decompose a data into primitive frequency elements. It reveals the data as a total of cosine functions fluctuating at separate frequencies [55]. Usually, the DCT is applied to the data to get the DCT coefficients which are split into two groups [56,57]; low frequencies are known as DC coefficients, and high frequencies are known as AC coefficients. High frequencies illustrate edge, details, and tiny changes [57], while low frequencies are linked with the brightness situations. The dimension of the DCT coefficient matrix is identical to the input data [58].
- **PCA** is a popular feature reduction approach that is commonly employed to compress the huge dimension of features via operating a covariance analysis among observed features. The PCA lessens the full number of observed variables to a reduced quantity of principal components. Such principal components resemble the variance of the original features. It is generally utilized if the observed features of a dataset are very correlated. The PCA is appropriate for datasets having very huge dimensions [59].

#### 3.3.5. Classification

**Figure 6:**
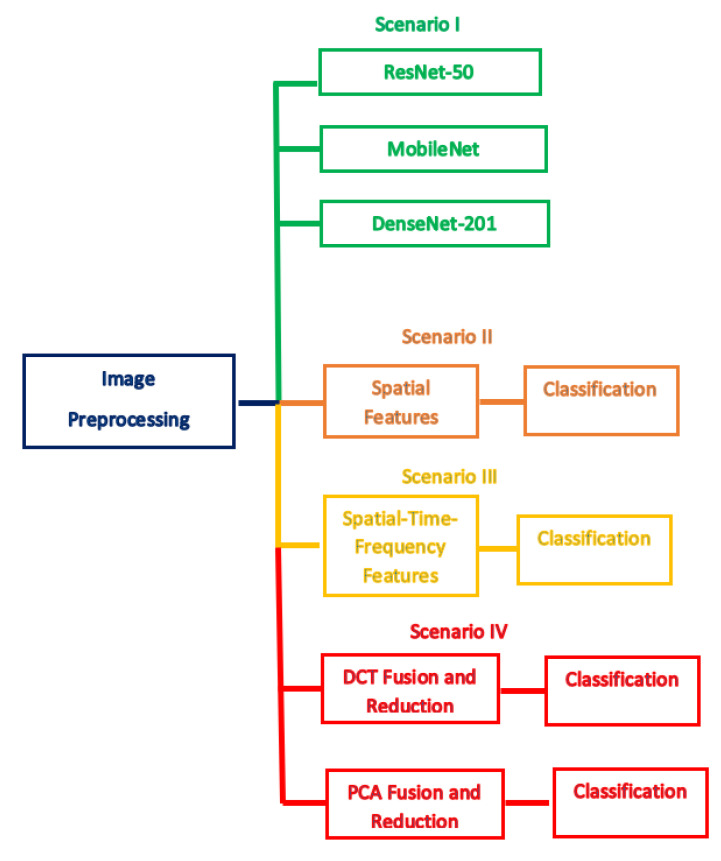
The four scenarios of MB-AI-His.

The classification procedure of this stage is done with four distinct scenarios. The initial scenario introduces the utilization of three deep pre-trained networks with transfer learning including ResNet-50, DenseNet-201, MobileNet CNNs as classifiers (end to end deep learning process). The second scenario represents the classification using the spatial features extracted in the spatial feature extraction stage of MB-AI-His. Later, in the third scenario, the classification process is achieved using the spatial-time-frequency features extracted in the time-frequency feature extraction stage of MB-AI-His. Finally, in the last scenario, the spatial-time-frequency features are fused using DCT and PCA and utilized to perform the classification process. Note that in this scenario the numbers of DCT coefficients and principal components are chosen using a sequential forward strategy to reduce the huge dimension of features. Five popular classifiers are used to perform the classification procedure including linear SVM, cubic SVM, k-nearest neighbors k-NN, linear discriminant analysis (LDA), and ensemble subspace discriminant (ESD). Figure 6 describes the four scenarios of the proposed MB-AI-His.

## 4. Experimental Setup

### 4.1. Parameters Setting

Initially, the FC layer of the pre-trained CNNs is modified to the number of classes of the childhood MB dataset (2 in the case of binary level and 4 for multiclass level) instead of the 1000 classes of ImageNet. Next, several parameters are altered for the three CNNs including the number of epochs, initial learning rate, mini-batch size, and validation frequency. The total amount of epochs and the initial learning rate are 20 and 3 × 10^−4^ respectively. The mini-batch size and validation frequency are 4 and 17 for binary class and 26 for multi-class, whereas the other CNN parameters are kept unchanged. The optimization algorithm used is the Stochastic Gradient Descent with Momentum (SGDM). To test the capability of the classification models, 5-fold cross-validation is utilized and repeated 5 times. For the k-NN classifier, the number of k is equal to 1 and the Euclidean distance is used as a distance metric, and these parameters attained the highest performance. For the ESD classifier, the number of learners is 30 and the subspace dimension is 1024.

### 4.2. Evaluation Metrics

To evaluate the performance of the introduced MB-AI-His, different evaluation metrics are employed. These metrics are the accuracy, the precision, the sensitivity, and the specificity. They are calculated using the following formulas [26] (1–4).

- True Positives (TP): Images that have their true label as positive and whose class is correctly classified to be positive.
- False Positives (FP): Images that have their true label as negative and whose class is wrongly classified to be positive.
- True Negatives (TN): Images that have their true label as negative and whose class is precisely classified to be negative.
- False Negatives (FN): Images that have their true label as positive and whose class is wrongly classified to be negative.

The accuracy is a performance metric that shows how the system has properly classified the childhood MB class and its four subtypes. Thus, it identifies the ability of the MB-AI-His to perform well.

(1) $$Accuracy=\frac{TP+TN}{TN+FP+FN+TP}\;\;\;\;\;\;$$

The sensitivity is for a given class, the number of images that are correctly classified as positive out of the sum of actual positives images.

(2) $$Sensitivity=\frac{TP}{TP+FN}\;\;\;\;\;\;\;\;\;\;$$

The specificity is for a given class, the number of images that are correctly classified as negative out of the sum of actual negative images.

(3) $$Specificity=\frac{TN}{TN+FP}\;\;\;\;\;\;\;$$

The precision is the proportion of images that are correctly classified as positive to the total number of images that are truly labeled to be positive.

(4) $$Precision=\frac{TP}{TP+FP}\;\;\;\;\;\;\;$$

## 5. Results

This section illustrates the classification results of the four scenarios of MB-AI-His. As mentioned before, MB-AI-His performs two levels of classification. The first level classifies the pediatric MB images as either normal or abnormal (binary classification). The other level classifies the four subtypes of MB (multi-class classification). Scenario I is an end-to-end deep learning procedure where ResNet-50, DenseNet-201, and MobileNet CNNs are used to perform the classification task. Scenario II resembles the extraction of the spatial features from the three-deep learning CNNs and using them individually to feed five classifiers including linear SVM, cubic SVM, LDA, and KNN, and ESD classifiers. Scenario III represents the extraction of the time-frequency features from the spatial DL features to form three spatial-time-frequency DL features sets. These feature sets are used individually for the classification process achieved by the same five classifiers. This scenario is executed to examine if the spatial-time-frequency feature set of a reduced dimension performs better than the spatial features alone. Scenario IV presents the fusion of the three spatial-time-frequency DL feature sets using DCT and PCA and using the reduced fused feature set to perform the classification process. Note that the numbers of DCT coefficients and principal components are selected using a sequential forward search strategy. This scenario is done to merge the benefits of the DL techniques and textural analysis feature extraction methods as well as combining the privilege of each CNN architecture. The scenario examines if this feature fusion successfully enhances the performance of MB-AI-His. It also investigates if DCT and PCA can produce a time-efficient CADx system with enhanced accuracy.

### 5.1. Scenario I Results

The classification performance of the three CNNs used to perform the end-to-end deep learning procedure for both binary and multi-class classification levels is shown in Table 6. The table shows that the classification accuracies achieved for the binary classification level are 100%, 90%, and 100% for the ResNet-50, MobileNet, and DenseNet-201 CNNs, respectively, whereas the training execution times are 2 min 5 s, 2 min 13 s, and 9 min for the ResNet-50, MobileNet, and DenseNet-201 CNNs, respectively. This means that the ResNet-50 CNN is faster than the DenseNet-201 CNN while achieving the same accuracy. For the multi-class classification level, the classification accuracies attained are 93.62%, 91.49%, and 89.36% for the ResNet-50, MobileNet, and DenseNet-201 CNNs, respectively. These accuracies indicate that the ResNet-50 CNN has the highest performance, followed by the MobileNet and DenseNet-201 CNNs. The training execution times are 4 mins 9 s, 2 mins 5 s, and 14 mins 10 s for the ResNet-50, MobileNet, and DenseNet-201 CNNs, respectively. 

### 5.2. Scenario II Results

The classification performance of the five classifiers trained with the spatial features extracted from each of the deep learning CNNs for both binary and multi-class classification levels is shown in Table 7. Table 7 indicates that for the binary classification level, the spatial DL features extracted from the ResNet 50 CNN and used to train the cubic SVM and LDA classifiers, the highest accuracy of 100% is achieved. Whereas, for the spatial DL features extracted from the DensNet-201 CNN and utilized to train the ESD classifier, a peak accuracy of 99.2% is attained. For the spatial DL features extracted from the MobileNet CNN, a maximum accuracy of 99.4% is obtained using the ESD classifier. On the other hand, for the multi-class classification level, the spatial DL features extracted from the ResNet 50 CNN and employed as inputs to the LDA classifier, the highest accuracy of 95.74% is acheived. Whereas, for the spatial DL features extracted from the DensNet-50 CNN, the LDA classifier attained a peak accuracy of 97.16%. While, for the spatial DL features extracted from MobileNet CNN, a maximum accuracy of 94.54% is obtained using the LDA classifier. These accuracies conclude that the LDA classifier outperforms all other classifiers and is suitable to classify the four subtypes of pediatric MB.

### 5.3. Scenario III Results

The accuracies obtained using the five classifiers learned with the spatial-time-frequency DL features extracted from each deep learning CNNs for both binary and multi-class classification levels are shown in Table 8. Table 8 demonstrates that for the binary classification level, the spatial-time-frequency DL features (1074 features) pulled out from the ResNet-50 CNN and used to build the LDA classifier achieved the highest accuracy of 100%, which is the same accuracy of the spatial features (2048 features) extracted from ResNet-50 as shown in Table 7 but with lower dimension. For the spatial-time-frequency DL features (1010 features) pulled out from the DensNet-50 CNN and utilized to learn the ESD classifier attained a peak accuracy of 99.2%, which is the same accuracy obtained by the same classifier when trained with the spatial features (1920 features) extracted from the DensNet-201 CNN (shown in Table 7) but with lower dimension. For the spatial-time-frequency DL features (660 features) extracted from the MobileNet CNN, a maximum accuracy of 98.4% is obtained using the ESD classifier. On the other hand, in the case of the multi-class classification level, for the spatial-time-frequency DL features (1074 features) extracted from the ResNet 50 CNN, the LDA classifier achieved the highest accuracy of 96.66%, which is higher than the 95.74% (shown in Table 7) obtained with the same classifier trained with only spatial DL features of higher dimension extracted from the ResNet-50 CNN. Whereas, for the spatial-time-frequency DL features (1010) extracted from the DensNet-201 CNN, the LDA classifier attained a peak accuracy of 98.46%, which is better than the 97.16% (shown in Table 7) obtained with the same classifier when trained with spatial features only which have a higher dimension of (1920 features). While, for the spatial-time-frequency DL features (690) extracted from the MobileNet CNN, a maximum accuracy of 98.46% is obtained using the LDA classifier which is better than the 94.54% (shown in Table 7) achieved using the same classifier trained with spatial features only of higher dimension (1280 features) extracted from the MobileNet CNN. These accuracies conclude that the spatial-time-frequency DL features are better than using the spatial DL features alone, as the spatial-time-frequency DL features have improved the classification accuracy and reduced the feature space dimension used in MB-AI-CADx. This makes them more appropriate to be used for classifying the four subtypes of pediatric MB. 

### 5.4. Scenario IV Results

This section illustrates the performance of the five classifiers used in MB-AI-His after the fusion process accomplished using both the PCA and DCT methods. It also describes the numbers of the DCT coefficients and principal components selected to reduce the feature space dimension and produce an efficient CADx. Table 9 shows the numbers of the DCT coefficients and principal components as well as classification accuracy (%) for the five classifiers used in MB-AI-His after fusion using the PCA and DCT approaches for the binary classification level. It is obvious from the table that both DCT and PCA have successfully enhanced the classification accuracy after the fusion process to reach 100% for all classifiers, which is higher than those obtained using the five classifiers trained with the individual spatial-time-frequency DL features shown in Table 8. Moreover, the numbers of DCT coefficients and principal components attained are 300 and 2 for the DCT and PCA, respectively, which are much lower than the 2274 features equivalent to the total sum of features of the spatial-time-frequency DL features extracted from the three CNNs.

**Figure 7:**
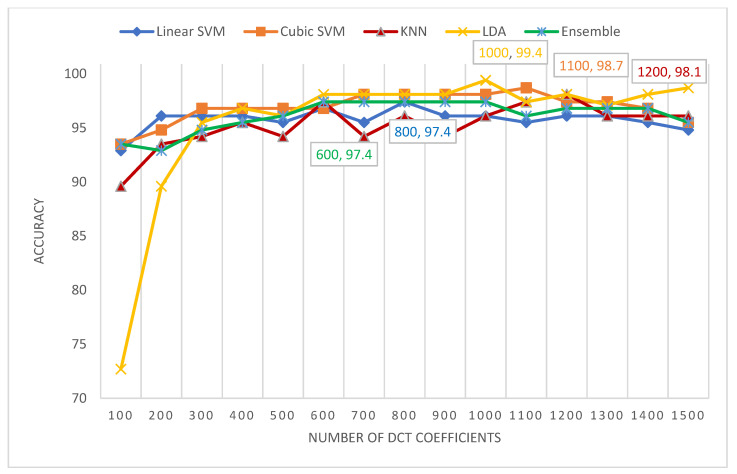
The number of DCT coefficients versus the classification accuracies attained for the five classifiers of MB-AI-His.

Figure 7 shows the number of DCT coefficients versus the classification accuracies attained for the five classifiers of MB-AI-His CADx. It is clear from Figure 7 that for the multi-class classification level, the highest accuracy of 99.4 % is attained using the LDA classifier using 1000 coefficients only, which is lower than the 2774 features of the fused spatial-time-frequency DL features of the three networks. Following the LDA classifier’ performance is the cubic SVM classifier, which attained an accuracy of 98.7% with 1100 DCT coefficients, followed by the k-NN classifier achieving an accuracy of 98.1% with 1200 DCT coefficients, ending by the linear SVM and ensemble (ESD) classifiers which achieved an accuracy of 97.4% using 800 and 600 coefficients, respectively.

**Figure 8:**
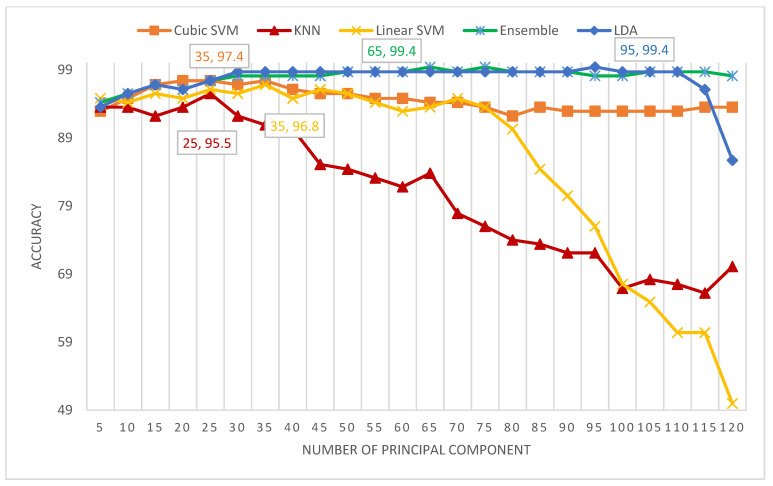
The number of principal components versus the classification accuracies attained for the five classifiers of MB-AI-His-CADx.

Figure 8 shows the number of principal components versus the classification accuracies attained for the five classifiers of MB-AI-His CADx. The figure indicates that the maximum accuracy of 99.4% is obtained using the LDA and ESD classifiers using only 95 and 65 principal components respectively. This performance is followed by the cubic SVM achieving an accuracy of 97.4% using 35 components, the linear SVM obtaining an accuracy of 96.8% using 35 components, and finally the k-NN attaining an accuracy of 95.5% using 25 components.

**Figure 9:**
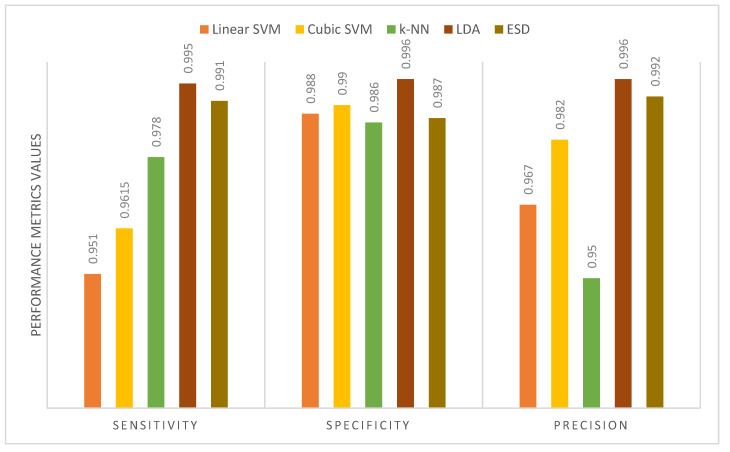
The performance metrics for the five classifiers used in MB-AI-His CADx after the fusion using PCA for the multi-class classification level.

**Figure 10:**
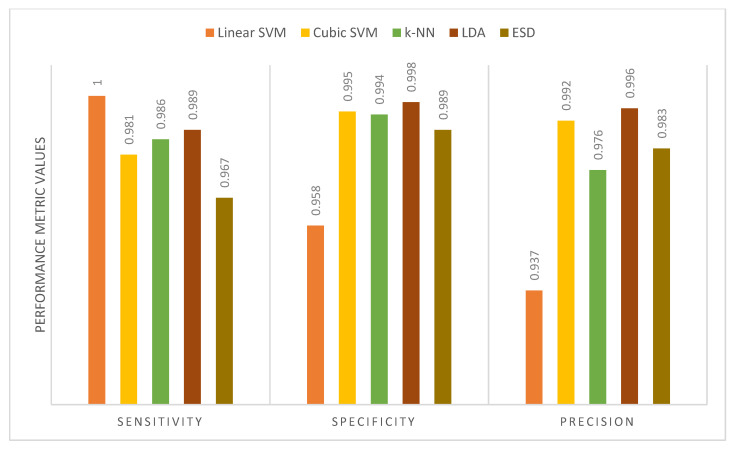
The performance metrics for the five classifiers used in MB-AI-His CADx after the fusion using DCT for the multi-class classification level.

Table 10 shows the performance metrics for the five classifiers used in MB-AI-His after the fusion process using the PCA and DCT methods for the binary classification level. It is obvious from the table that the sensitivities, specificities, and precisions are equal to 1 for all classifiers. This is because MB-AI-His is capable of perfectly differentiating between normal images and images of childhood MB achieving an accuracy of 100% using the k-NN, linear and cubic SVM, the LDA, and ESD classifiers. In other words, the combination of features used in MB-AI-His is capable of discriminating among normal and abnormal images, enabling the five classifiers to attain 100% accuracy. Figure 9 shows the performance metrics for the five classifiers used in MB-AI-His CADx after the fusion process using the PCA approach for the multi-class classification level. The figure indicates that the maximum sensitivity, specificity, and precision of 0.995, 0.996, and 0.996 are attained using the LDA classifier. Figure 10 shows the performance metrics for the five classifiers used in MB-AI-His CADx after the fusion procedure using the DCT method for the multi-class classification level. The figure indicates that the highest specificity and precision are attained using the LDA classifier. For medical systems to be reliable, the specificity and precision should be greater than 0.95, whereas the sensitivity should be greater than 0.8 as indicated in [60,61]. It is clear from Table 10 and Figure 9 and Figure 10 that sensitivities for the binary and multi-class levels are greater than 0.8. The specificities and precisions are also greater than 0.95 for both the binary and multi-class classification levels, therefore, MB-AI-His can be considered as a reliable CADx system that enables the accurate and reliable diagnosis of pediatric MB and its subtypes.

Table 11 shows the training execution time for the five classifiers of MB-AI-His after the fusion procedure done using the DCT and PCA methods for both the binary and multi-class classification levels compared to the end-to-end DL process. The table proves that the fusion process using both the PCA and DCT methods has efficiently reduced the training execution time for both the binary and multi-class classification levels. This is clear as for the binary classification level, the lowest training execution times are 1.996 s and 0.858 s for thePCA and DCT obtained using the LDA and k-NN classifiers respectively, which attained 100% accuracy. These execution times are much lower than those of 125 s, 132 s, and 540 s obtained using the ResNet-50, MobileNet, and DenseNet-201 CNNs, respectively. On the other hand, for the multi-class classification level, the training execution times for the LDA classifiers are 2.79 s and 2 s for the PCA and DCT approaches, where they obtained the highest accuracy of 99.4%. These execution times are much lower than those of 249 s,125 s, and 850 s obtained using the ResNet-50, MobileNet, and DenseNet-201 CNNs, respectively.

## 6. Discussion

MB is the utmost common childhood malignant brain tumor [4]. It is the main reason for cancer-related disease and mortality among children [5,6]. Correct identification of the pediatric MB and its subtypes can lead to an increased 2 and 5 year survival rate as described in [8]. Since follow-up medication extremely depends on identifying the subtype of MB, it is essential to achieve an accurate diagnosis [13]. MRI imaging modality produces insufficient accuracy when classifying the subtypes of MB, whereas the histopathological investigation of biopsy samples is more capable in accurately diagnosing the childhood MB and its subtypes [11]. However, the manual analysis of histopathological is very time consuming, hard, and requires a need for a pathologist with great experience and skills to assess the very detailed property of the subtypes of MB. The availability of such pathologists is smaller than the number of patients, especially in the developed and developing countries. Due to this lack of availability patients travel abroad to make such analyses for better prospects which is exhausting and expensive [13]. To overcome these challenges, the automatic diagnosis using CADx systems are recommended. These systems could assist pathologists in the automatic analysis of histopathological images, thus decreasing the cost of diagnosis [22]. 

This paper proposes a CADx system, namely MB-AI-His, to automatically diagnose the pediatric MB and its subtypes from histopathological images with high accuracy and efficient time. MB-AI-His consists of five stages: the image preprocessing, subsequent by spatial feature extraction, time-frequency feature extraction, feature fusion and reduction, and finally the classification stage. Images are augmented and resized in the preprocessing stage. Next, spatial DL features are extracted from the ResNet-50, MobileNet, and DenseNet-201 CNNs in the spatial feature extraction stage. Afterward, time-frequency features are extracted using the DWT approach from the spatial DL features of the previous stage to form three spatial-time-frequency DL features. Then, these features are fused using the DCT and PCA methods to produce a time-efficient system. Finally, the classification stage is made via four different scenarios. Initially, the pre-trained ResNet-50, MobileNet, and DenseNet-201 CNNs are trained in an end-to-end classification process which corresponds to the first scenario. Next, spatial features are pulled out and used individually to train five machine learning classifiers corresponding to the second scenario. Afterward, in the third scenario, the spatial-time-frequency DL features (which have a lower dimension than the spatial features) are utilized individually to learn the five classifiers. Finally, in the last scenario, those features are fused using PCA and DCT which further reduce the dimension of the features to produce a timely efficient system.

**Figure 11:**
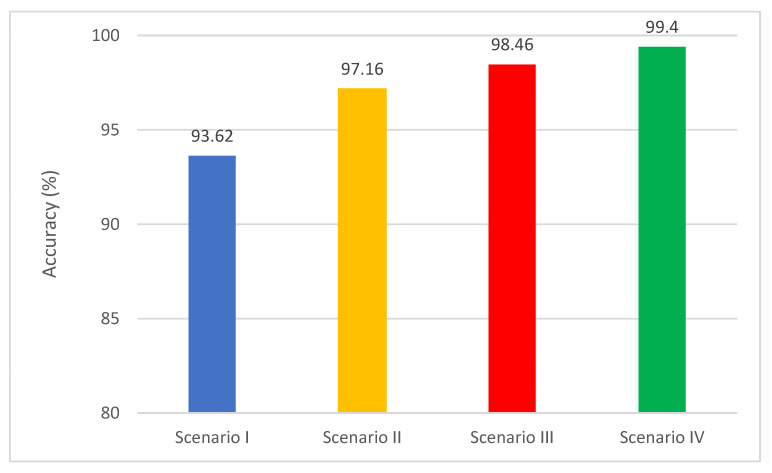
A comparison between the highest classification accuracy achieved for each scenario for multi-class classification level.

Figure 11 shows a comparison between the highest classification accuracy achieved for each scenario for the multi-class classification level. The figure verifies that each scenario enhances the accuracy of MB-AI-His compared to the previous scenario. This means that using spatial features with ML classifiers (scenario II) is better than the end-to-end DL process of scenario I. Using spatial-time-frequency DL features (Scenario III) is also better than using only spatial features. Finally, fusing spatial-time-frequency with the PCA method (Scenario IV) is superior to using the individual features of the three former scenarios. 

**Figure 12:**
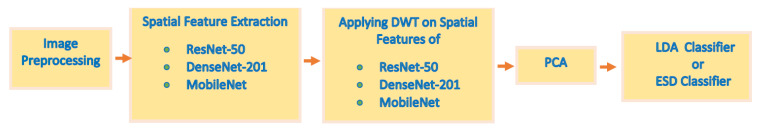
The final architecture of MB-AI-His.

For the binary classification level, the spatial-time-frequency features extracted from the MobileNet, DenseNet-201, and ResNet-50 CNNs followed by the DWT method are reduced using both the PCA and DCT methods. The PCA and DCT feature reduction methods have attained an accuracy of 100% for the five classifiers used in MB-AI-His, as shown in Table 9. On the other hand, for the multi-class classification level, the PCA methods has reduced those spatial-time-frequency features extracted from the three CNNs and the DWT approach and led to an accuracy of 99.4% using the LDA and ESD classifiers. Thus, the architecture of MB-AI-His for both the binary and multi-class classification levels can be concluded as shown in Figure 12. This figure shows that MB-AI-His architecture represents the fusion of the MobileNet, DenseNet-201, and ResNet-50 CNN features after applying the DWT method to each spatial feature individually. Afterward, these fused features are reduced using the PCA method and then classified via the LDA or ESD classifier.

All experiments were performed using Matlab 2020 a. The processor used is Intel(R) Core (TM) i7-10750H (10^th^ generation), processor frequency of 2.6 GHz, Hexa-core processor RAM 16 GB of type DDR4, hard disc capacity of 1.512 TB, and 64-bit operating system. The video controller is NVIDIA GeForce GTX 1660, graphics card capacity is 6 GB.

To verify the completeness of the introduced MB-AI-His CADx, it is compared with related CADx based on the same dataset. This comparison is shown in Table 12. The table proves the competence of MB-AI-His CADx over other related CADx for both the binary and multi-class classification levels. This is because MB-AI-His CADx achieved an accuracy of 100%, which is similar to that obtained by [7,31,33], but higher than that obtained by [32]. The competence of MB-AI-His appears clearly in classifying the four subtypes of childhood MB, as it attained an accuracy of 99.4%, a sensitivity of 0.995, a specificity of 0.996, and a precision of 0.996, which are higher than all the related CADx. MB-AI-His is reliable for both the binary and multi-class classification levels which is not the case in other studies. Therefore, it can be used to help doctors and pathologists in achieving an accurate diagnosis, thus reducing the cost of diagnosis and reduce the misdiagnosis that might cause during the manual diagnosis by a pathologist. It can also fasten the diagnosis procedure and reduce other challenges regarding manual diagnosis.

## 7. Conclusions

This paper proposed a time-efficient CADx, namely MB-AI-His, for automatic diagnosis of pediatric MB and its subtypes from histopathological images. It consists of image processing, spatial feature extraction, time-frequency feature extraction, feature fusion and reduction, and classification stages. Spatial DL features were extracted from ResNet-50, MobileNet, and DenseNet-201 CNNs in the spatial feature extraction stage. Afterward, spatial-time-frequency DL features were extracted from spatial DL features using DWT. Next, these three sets of features were merged using PCA and DCT feature reduction methods. MB-AI-His performed the classification of MB and its subtype using four different scenarios. Scenario I used the CNNs to perform classification. Spatial DL features were extracted from the three CNNS and used individually to train five ML classifiers in scenario II. Spatial-time-frequency DL features extracted in the time-frequency feature extraction stage were utilized individually to train the five ML classifiers in scenario III. Finally, these feature sets were combined using PCA and DCT and employed to train the five ML classifiers. The results showed that each scenario has improved the classification accuracy, and this appeared clearly in classifying the four subtypes of MB. The results of scenario III showed that using spatial-time-frequency was better than using spatial features alone (scenario II) and (scenario I). Moreover, fusing such features using PCA and DCT was superior and achieved accuracies of 100% and 99.4% for binary and multi-class classification levels respectively, which are higher than scenario III and scenario II and could extremely reduce the training execution time compared to scenario I. This means that MB-AI-His is accurate, reliable, and time-efficient. It can be used by the pathologist to reduce the complications they face while analyzing histopathological images. It can also speed up the diagnosis and make it more accurate which will correspondingly lower the cost of diagnosis, reduce the risk of tumor progression, and help in choosing the appropriate follow-up and treatment plans. Future work will consider collecting additional data from more patients and making a full dataset available for researchers. Further investigation will be conducted on using more DL methods to analyze childhood MB subtypes.

## Figures and Tables

**Table 1:** A list of related studies that used histopathological images along with their limitations.

Article	Segmentation	Features	Classifier	Accuracy	Medulloblastoma (MB) Class	Limitations
[16]	N/A	TICA ^1^ Wavelet analysis 2-layered convolutional neural networks (CNN)	Soft-max	99.7%	Anaplastic and Non- anaplastic	Use only one type of feature extraction either textural features or spatial features extracted from CNN. Very small dataset (10 images only)
[28]	N/A	TICA	Soft-max	97%	Anaplastic and Non- anaplastic	Depends on only texture-based feature extractors. Very small dataset (10 images only)
[12]	N/A	Haar Wavelet Transform	k-NN ^2^	87%	Anaplastic and Non- anaplastic	Use only one type of feature extraction (textural features) to build their computer-aided diagnosis (CADx) Very small dataset (10 images only)
[29]	N/A	Haar Haralick Laws textural features	RF ^3^	91%	Anaplastic and Non- anaplastic	Use an only individual type of feature extraction to build their CADx Very small dataset (6 images only)
[30]	N/A	16-layered CNN 2-Layered CNN	softmax	76.6% 89.8%	Anaplastic and Non- anaplastic	Depends on only spatial deep learning-based feature extractors. Very small dataset (10 images only)
[7]	K-means clustering	HOG ^4^ GLCM ^5^ GLRM ^6^ Tamura Color Feature LBP ^7^ Morphological Principal component analysis (PCA)	SVM	84.9%	Classic Desmoplastic Nodular Large Cell (anaplastic)	Depends only on conventional handcrafted features. They used only individual feature set to perform the classification task.
[31]	K-means clustering	HOG GLCM rGLRM Tamura Color Feature LBP Morphological MANOVA ^8^	SVM	65.2%	Classic Desmoplastic Nodular Large Cell (anaplastic)	Depends only on conventional handcrafted features. Used only individual feature set to perform the classification task.
[13]	K-means clustering	Different combinations of fused features including: HOG GLCM GLRM Tamura LBP PCA	SVM	96.7%	Classic Desmoplastic Nodular Large Cell (anaplastic)	Depends only on conventional handcrafted features.
[32]	N/A	AlexNet	Softmax	79.3%	Classic Desmoplastic Nodular Large Cell (anaplastic)	Depends only on spatial deep learning (DL) features.
		VGG-16 ^10^		65.4%		
[32]	N/A	AlexNet DL features	SVM	93.21%	Classic Desmoplastic Nodular Large Cell (anaplastic)	Depends only on spatial DL features. Use Individual DL for the classification task.
		VGG-16 DL features		93.38%		

^1^ TICA: Topographic independent component analysis, ^2^ k-NN: k-nearest neighbors,^3^ RF: Random Forest, ^4^ HOG: Histogram of oriented gradients, ^5^ GLCM: grey level covariance matrix, ^6^ GLRM: grey level run matrix, ^7^ LBP: local binary pattern, ^8^ MANOVA: Multivariate analysis of variance, and ^10^ VGG: Visual Geometry Group.

**Table 2:** The dimensions of the various layers of ResNet 50 CNN.

Layer Label	Input Layer Dimension	Output Dimension
Input Layer		224 × 224 × 3
Conv1	112 × 112 × 64	Filter size = 7 × 7 Number of filters = 64 Stride = 2 Padding = 3
pool1	56 × 56 × 64	Pooling size = 3 × 3 Stride = 2
conv2_x	56 × 56 × 64	$\left[\begin{array}{cc} 1\times 1 & 64\\ 3\times 3 & 64\\ 1\times 1 & 256 \end{array}\right]$ ×3
conv3_x	28 × 28 × 128	$\left[\begin{array}{cc} 1\times 1 & 128\\ 3\times 3 & 128\\ 1\times 1 & 512 \end{array}\right]$ ×4
conv4_x	14 × 14 × 256	$\left[\begin{array}{cc} 1\times 1 & 256\\ 3\times 3 & 256\\ 1\times 1 & 1024 \end{array}\right]$ ×6
conv5_x	7 × 7 × 512	$\left[\begin{array}{cc} 1\times 1 & 512\\ 3\times 3 & 512\\ 1\times 1 & 2048 \end{array}\right]$ ×3
Average pooling		Pool size = 7 × 7 Stride = 7
		1 × 1
Fully connected (FC) Layer		1000

**Table 3:** The dimensions of the various layers of DenseNet-201 CNN.

Layer Label	Input Layer Dimension	Output Dimension
Input Layer		224 × 224 × 3
Convolution	112 × 112	Filter size = 7 × 7 Stride = 2 Padding = 3
pooling	56 × 56	Maximum Pooling = 3 × 3 Stride = 2
Dense Block 1	56 × 56	$\left[\begin{array}{cc} 1 & \times \;\;\;1\\ 3 & \times \;\;\;3 \end{array}\right]\times 6$
Transition Layer 1	56 × 56	1 × 1 convolution
	28 × 28	2 × 2 average pooling, stride =2
Dense Block 2	28 × 28	$\left[\begin{array}{cc} 1 & \times \;\;\;1\\ 3 & \times \;\;\;3 \end{array}\right]\times 12$
Transition Layer 2	28 × 28	1 × 1 convolution
	14 × 14	2 × 2 average pooling, stride =2
Dense Block 3	14 × 14	$\left[\begin{array}{cc} 1 & \times \;\;\;1\\ 3 & \times \;\;\;3 \end{array}\right]\times 48$
Transition Layer 3	14 × 14	1 × 1 convolution
	7 × 7	2 × 2 average pooling, stride =2
Dense Block 4	7 × 7	$\left[\begin{array}{cc} 1 & \times \;\;\;1\\ 3 & \times \;\;\;3 \end{array}\right]\times 32$
Pooling		Average Pooling= 7 × 7 Stride = 7
		1 × 1
FC Layer		1000

**Table 4:** The general structure of MobileNet CNN.

Layer Label	Input Layer Dimension	Filter and Stride Size
Convolution/S2	224 × 224 × 3	Filter size = 3 × 3 × 3 × 32 Stride = 2
Convolution/dw^8^/S1	112 × 112 × 32	Filter size = 3 × 3 × 32 dw Stride = 1
Convolution/S1	112 × 112 × 32	Filter size 1 × 1 × 32 ×64 Stride = 1
Convolution/dw/S2	112 × 112 × 64	Filter size = 3 × 3 × 64 dw Stride = 2
Convolution/S1	56 × 56 × 64	Filter size 1 × 1 × 64 × 128 Stride = 1
Convolution/dw/S1	56 × 56 × 128	Filter size = 3 × 3 × 128 dw Stride = 2
Convolution/S1	56 × 56 × 128	Filter size 1 × 1 × 128 × 128 Stride = 1
Convolution/dw/S2	56 × 56 × 128	Filter size = 3 × 3 × 128 dw Stride = 2
Convolution/S1	28 × 28 × 128	Filter size 1 × 1 × 128 × 256 Stride = 1
Convolution/dw/S1	28 × 28 × 256	Filter size = 3 × 3 × 256 dw Stride = 1
Convolution/S1	28 × 28 × 256	Filter size 1 × 1 × 256 × 256 Stride = 1
Convolution/dw/S2	56 × 56 × 128	Filter size = 3 × 3 × 256 dw Stride = 2
Convolution/S1	14 × 14 × 256	Filter size 1 × 1 × 256 × 512 Stride = 1
5 × Convolution dw/S1 5 × Convolution S1	14 × 14 × 512 14 × 14 × 512	Filter size = 3 × 3 × 512 dw Filter size 1 × 1 × 512 × 512 Stride = 1
Convolution dw/S2	14 × 14 × 512	Filter size = 3 × 3 × 512 dw Stride 2
Convolution/S1	7 × 7 × 512	Filter size 1 × 1 × 512 × 512 Stride = 1
Convolution dw/S2	7 × 7 × 1024	Filter size = 3 × 3 × 1024 dw Stride 2
Convolution/S1	7 × 7 × 1024	Filter size 1 × 1 × 1024 × 1024 Stride = 1
Pooling		Average Pooling= 7 × 7 Stride = 1
		1 × 1 × 1024
FC Layer		1 ×1 × 1000

dw stands for depthwise.

**Table 5:** The number of layers and output size of each CNN.

CNN Structure	Number of Layers	Size of Output (Features)
**ResNet-50**	50	2048
**MobileNet**	19	1280
**DenseNet-201**	201	1920

**Table 6:** The classification testing accuracy (%) and execution training time for the three CNNs for both binary and multi-class classification.

CNN Structure	Binary Classification Level		Multi-Class Classification Level	
	Accuracy (%)	Execution Time	Accuracy (%)	Execution Time
**ResNet-50**	100	2 min 5 s	93.62	4 min 9 s
**MobileNet**	90	2 min 13 s	91.49	2 min 5 s
**DenseNet-201**	100	9 min	89.36	14 min 10 s

**Table 7:** The classification testing accuracy (%) for the five classifiers used in MB-AI-His trained using spatial DL features extracted from the three CNNs.

Binary Classification Level					
Features	Linear-SVM	Cubic-SVM	k-NN	Linear Discriminant Analysis(LDA)	Ensemble Subspace Discriminant(ESD)
**Spatial-ResNet-50**	99.6	100	98	100	94.8
**Spatial-MobileNet**	99.2	99.2	98.2	99	99.4
**Spatial-DenseNet-201**	98	98	97.6	98	99.2
**Multi-Class Classification Level**					
**Spatial-ResNet-50**	93.66	94.96	88.18	95.74	93.9
**Spatial-MobileNet**	91.72	92.2	87.28	94.54	93.4
**Spatial-DenseNet-201**	94.32	96.26	92.88	97.16	94.84

**Table 8:** The classification testing accuracy (%) for the five classifiers used in MB-AI-His trained using spatial-time-frequency DL features extracted from the three CNNs.

Binary Classification Level					
Features	Linear-SVM	Cubic-SVM	k-NN	LDA	ESD
**Spatial-Time-Frequency-ResNet-50**	100	100	98.2	100	99
**Spatial-Time-Frequency-MobileNet**	98.2	98.4	98.4	98	98.2
**Spatial-Time-Frequency-DenseNet-201**	98.4	98.2	98.4	98.2	99.2
**Multi-Class Classification Level**					
**Spatial-Time-Frequency-ResNet-50**	94.32	95.22	89.48	96.66	95.36
**Spatial-Time-Frequency-MobileNet**	93.76	94.18	90.14	94.32	93.66
**Spatial-Time-Frequency-DenseNet-201**	95.74	97.16	95.08	98.46	97.42

**Table 9:** The numbers of discrete cosine transform (DCT) and principal components, and classification testing accuracy (%) for the five classifiers used in MB-AI-His after the fusion using PCA and DCT for the binary classification level.

	Binary Classification Level					
Features	No of Features	Linear-SVM	Cubic-SVM	k-NN	LDA	ESD
**DCT**	300	100	100	100	100	100
**PCA**	2	100	100	100	100	100

**Table 10:** The performance metrics for the 5 classifiers used in MB-AI-His CADx after the fusion using PCA and DCT for binary class classification level.

Binary Classification Level					
Features	Linear-SVM	Cubic-SVM	k-NN	LDA	ESD
DCT and PCA					
**Sensitivity**	1	1	1	1	1
**Specificity**	1	1	1	1	1
**Precision**	1	1	1	1	1

**Table 11:** The training execution time (sec) of the three CNN used in MB-AI-His CADx and after the fusion stage of MB-AI-His CADx for both binary and multi-class classification levels.

			Multi-Class Classification Level				
DL Method	Time	Feature Reduction Method	Linear-SVM Time	Cubic-SVM Time	k-NN Time	LDA Time	ESD Time
**ResNet-50**	249	**DCT**	2.15	2.03	1.98	2	3.39
**MobileNet**	125	**PCA**	2.74	3.17	4.75	2.79	6.23
**DenseNet-201**	850						
			**Binary-Class Classification Level**				
**DL Method**	**Time**	**Feature Reduction Method**	**Linear-SVM Time**	**Cubic-SVM Time**	**k-NN Time**	**LDA Time**	**ESD Time**
**ResNet-50**	125	**DCT**	0.873	0.886	0.858	0.888	2.54
**MobileNet**	132	**PCA**	2.05	2	2.07	1.996	3.314
**DenseNet-201**	540						

**Table 12:** A comparison between MB-AI-His and related CADx based on the same dataset.

Binary Classification Level					
Article	Method	Testing Accuracy (%)	Sensitivity	Specificity	Precision
[33]	GLCM, GRLN, HOG, Tamura, and LBP features++SVM	100	1	1	1
[7]	Color and Shape features+ PCA+ SVM	100	1	1	1
[31]	GLCM, GRLN, HOG, Tamura and LBP features++MANOVA+SVM	100	1	1	1
[32]	AlexNet VGG-16	98.5 98.12	-	-	-
[32]	AlexNet+SVM VGG-16+SVM	99.44 99.62	-	-	-
**Proposed MB-AI-His**	DenseNet + MobileNet +ResNet fusion using PCA+LDA or ESD classifier	100	1	1	1
**Multi-Class Classification Level**					
		**Testing Accuracy (%)**	**Sensitivity**	**Specificity**	**Precision**
[7]	Color and Shape features+ PCA+ SVM	84.9	-	-	-
[13]	LBP+GRLM+GLCM +Tamura features +SVM	91.3	0.913	0.97	0.913
[13]	LBP+GRLM+GLCM+Tamura features + PCA+SVM	96.7	-	-	-
[31]	GLCM, GRLN, HOG, Tamura and LBP features++MANOVA+SVM	65.21	0.72	-	0.666
[32]	AlexNet VGG-16	79.33 65.4	-	-	-
[32]	AlexNet+ SVM VGG-16+SVM	93.21 93.38	-	-	-
**Proposed MB-AI-His**	DenseNet+MobileNet+ResNet fusion using PCA+LDA or ESD classifiers	99.4	0.995	0.996	0.996

## Data Availability

The data presented in this study are openly available in [IEEE Dataport] at doi [10.21227/w0m0-mw21]: https://ieee-dataport.org/open-access/childhood-medulloblastoma-microscopic-images.

## References

[1] Grist J.T., Withey S., MacPherson L., Oates A., Powell S., Novak J., Abernethy L., Pizer B., Grundy R., Bailey S. (2020). Distin-guishing between Paediatric Brain Tumour Types Using Multi-Parametric Magnetic Resonance Imaging and Machine Learning: A Multi-Site Study. NeuroImage Clin..

[2] Bright C., Reulen R., Fidler M., Guha J., Henson K., Wong K., Kelly J., Frobisher C., Winter D., Hawkins M. (2015). Cerebro-vascular Complications in 208,769 5-Year Survivors of Cancer Diagnosed Aged 15–39 Years Using Hospital Episode Statistics: The Population-Based Teenage and Young Adult Cancer Survivor Study (TYACSS): Abstract O-24. Eur. J. Cancer Care.

[3] Dong J., Li L., Liang S., Zhao S., Zhang B., Meng Y., Zhang Y., Li S. (2020). Differentiation Between Ependymoma and Medullo-blastoma in Children with Radiomics Approach. Acad. Radiol..

[4] Ritzmann T.A., Grundy R.G. (2018). Translating Childhood Brain Tumour Research into Clinical Practice: The Experience of Molec-ular Classification and Diagnostics. J. Paediatr. Child Health.

[5] Pollack I.F., Jakacki R.I. (2011). Childhood brain tumors: Epidemiology, current management and future directions. Nat. Rev. Neurol..

[6] Iv M., Zhou M., Shpanskaya K., Perreault S., Wang Z., Tranvinh E., Lanzman B., Vajapeyam S., Vitanza N., Fisher P. (2018). MR Imaging–Based Radiomic Signatures of Distinct Molecular Subgroups of Medulloblastoma. Am. J. Neuroradiol..

[7] Das D., Mahanta L.B., Ahmed S., Baishya B.K., Haque I. (2018). Study on Contribution of Biological Interpretable and Comput-er-Aided Features towards the Classification of Childhood Medulloblastoma Cells. J. Med. Syst..

[8] Davis F.G., Freels S., Grutsch J., Barlas S., Brem S. (1998). Survival rates in patients with primary malignant brain tumors stratified by patient age and tumor histological type: An analysis based on Surveillance, Epidemiology, and End Results (SEER) data, 1973–1991. J. Neurosurg..

[9] Vicente J., Fuster-García E., Tortajada S., García-Gómez J.M., Davies N., Natarajan K., Wilson M., Grundy R.G., Wesseling P., Monleon D. (2013). Accurate classification of childhood brain tumours by in vivo 1H MRS—A multi-centre study. Eur. J. Cancer.

[10] Fetit A.E., Novak J., Rodriguez D., Auer D.P., Clark C.A., Grundy R.G., Peet A.C., Arvanitis T.N. (2017). Radiomics in paediatric neuro-oncology: A multicentre study on MRI texture analysis. NMR Biomed..

[11] Zarinabad N., Abernethy L.J., Avula S., Davies N.P., Gutierrez D.R., Jaspan T., MacPherson L., Mitra D., Rose H.E., Wilson M. (2017). Application of pattern recognition techniques for classification of pediatric brain tumors by in vivo 3T 1 H-MR spectroscopy—A multi-center study. Magn. Reson. Med..

[12] Cruz-Roa A., González F., Galaro J., Judkins A.R., Ellison D., Baccon J., Madabhushi A., Romero E. (2012). A Visual Latent Se-mantic Approach for Automatic Analysis and Interpretation of Anaplastic Medulloblastoma Virtual Slides. Proceedings of the International Conference on Medical Image Computing and Computer-Assisted Intervention.

[13] Das D., Mahanta L.B., Ahmed S., Baishya B.K. (2020). Classification of childhood medulloblastoma into WHO-defined multiple subtypes based on textural analysis. J. Microsc..

[14] Ellison D.W. (2010). Childhood medulloblastoma: Novel approaches to the classification of a heterogeneous disease. Acta Neuropathol..

[15] Pickles J.C., Hawkins C., Pietsch T., Jacques T.S. (2018). CNS embryonal tumours: WHO 2016 and beyond. Neuropathol. Appl. Neurobiol..

[16] Otálora S., Cruz-Roa A., Arevalo J., Atzori M., Madabhushi A., Judkins A.R., González F., Müller H., Depeursinge A. (2015). Combining Unsupervised Feature Learning and Riesz Wavelets for Histopathology Image Representation: Application to Identifying Anaplastic Medulloblastoma. Proceedings of the Constructive Side-Channel Analysis and Secure Design.

[17] Arevalo J., Cruz-Roa A., Gonzalez O F.A. (2014). Histopathology Image Representation for Automatic Analysis: A State-of-the-Art Review. Rev. Med..

[18] Dasa D., Mahantaa L.B., Baishyab B.K., Haqueb I., Ahmedc S. (2018). Automated Histopathological Diagnosis of Pediatric Me-dulloblastoma–A Review Study. Int. J. Appl. Eng. Res..

[19] Zhang X., Zhang Y., Qian B., Liu X., Li X., Wang X., Yin C., Lv X., Song L., Wang L., Rojas I., Valenzuela O., Rojas F., Ortuño F. (2019). Classifying Breast Cancer Histo-pathological Images Using a Robust Artificial Neural Network Architecture. Proceedings of the Bioinformatics and Bio-medical Engineering.

[20] Anwar F., Attallah O., Ghanem N., Ismail M.A. (2020). Automatic Breast Cancer Classification from Histopathological Images. Proceedings of the 2019 International Conference on Advances in the Emerging Computing Technologies (AECT).

[21] Robboy S.J., Weintraub S., Horvath A.E., Jensen B.W., Alexander C.B., Fody E.P., Crawford J.M., Clark J.R., Cantor-Weinberg J., Joshi M.G. (2013). Pathologist Workforce in the United States: I. Development of a Predictive Model to Examine Factors Influencing Supply. Arch. Pathol. Lab. Med..

[22] Kumar A., Singh S.K., Saxena S., Lakshmanan K., Sangaiah A.K., Chauhan H., Shrivastava S., Singh R.K. (2020). Deep feature learning for histopathological image classification of canine mammary tumors and human breast cancer. Inf. Sci..

[23] Ragab D.A., Sharkas M., Attallah O. (2019). Breast Cancer Diagnosis Using an Efficient CAD System Based on Multiple Classifiers. Diagnostics.

[24] Attallah O., Abougharbia J., Tamazin M., Nasser A.A. (2020). A BCI System Based on Motor Imagery for Assisting People with Motor Deficiencies in the Limbs. Brain Sci..

[25] Attallah O., Ma X. (2014). Bayesian neural network approach for determining the risk of re-intervention after endovascular aortic aneurysm repair. Proc. Inst. Mech. Eng. Part. H J. Eng. Med..

[26] Attallah O., Ragab D.A., Sharkas M. (2020). MULTI-DEEP: A novel CAD system for coronavirus (COVID-19) diagnosis from CT images using multiple convolution neural networks. PeerJ.

[27] Attallah O., Karthikesalingam A., Holt P.J., Thompson M.M., Sayers R., Bown M.J., Choke E.C., Ma X. (2017). Using multiple classifiers for predicting the risk of endovascular aortic aneurysm repair re-intervention through hybrid feature selection. Proc. Inst. Mech. Eng. Part. H J. Eng. Med..

[28] Cruz-Roa A., Arevalo J., Basavanhally A., Madabhushi A., Gonzalez F. (2015). A comparative evaluation of supervised and unsupervised representation learning approaches for anaplastic medulloblastoma differentiation. Proceedings of the 10th International Symposium on Medical Information Processing and Analysis.

[29] Lai Y., Viswanath S., Baccon J., Ellison D., Judkins A.R., Madabhushi A. (2011). A Texture-Based Classifier to Discriminate Ana-plastic from Non-Anaplastic Medulloblastoma. Proceedings of the 2011 IEEE 37th Annual Northeast Bioengineering Conference (NEBEC).

[30] Cruz-Roa A., Arévalo J., Judkins A., Madabhushi A., González F. (2015). A method for medulloblastoma tumor differentiation based on convolutional neural networks and transfer learning. Proceedings of the 11th International Symposium on Medical Information Processing and Analysis.

[31] Das D., Mahanta L.B., Ahmed S., Baishya B.K. (2020). A study on MANOVA as an effective feature reduction technique in classification of childhood medulloblastoma and its subtypes. Netw. Model. Anal. Health Inform. Bioinform..

[32] Das D., Mahanta L.B., Baishya B.K., Ahmed S. (2020). Classification of Childhood Medulloblastoma and its subtypes using Transfer Learning features—A Comparative Study of Deep Convolutional Neural Networks. Proceedings of the 2020 International Conference on Computer, Electrical & Communication Engineering (ICCECE).

[33] Das D., Mahanta L.B., Ahmed S., Baishya B.K., Haque I. (2019). Automated Classification of Childhood Brain Tumours Based on Texture Feature. Songklanakarin J. Sci..

[34] Das D., Lipi B. (2020). Mahanta Childhood Medulloblastoma Microscopic Images 2020.

[35] Mahmud M., Kaiser M.S., Hussain A., Vassanelli S. (2018). Applications of Deep Learning and Reinforcement Learning to Biological Data. IEEE Trans. Aerosp. Electron. Syst..

[36] Angermueller C., Pärnamaa T., Parts L., Stegle O. (2016). Deep learning for computational biology. Mol. Syst. Biol..

[37] Attallah O., Sharkas M.A., Gadelkarim H. (2020). Deep Learning Techniques for Automatic Detection of Embryonic Neurodevel-opmental Disorders. Diagnostics.

[38] Zemouri R., Zerhouni N., Racoceanu D. (2019). Deep Learning in the Biomedical Applications: Recent and Future Status. Appl. Sci..

[39] Baraka A., Shaban H., El-Nasr A., Attallah O. (2019). Wearable Accelerometer and SEMG-Based Upper Limb BSN for Tele-Rehabilitation. Appl. Sci..

[40] Ragab D.A., Attallah O. (2020). FUSI-CAD: Coronavirus (COVID-19) diagnosis based on the fusion of CNNs and handcrafted features. PeerJ Comput. Sci..

[41] Kawahara J., Brown C.J., Miller S.P., Booth B.G., Chau V., Grunau R.E., Zwicker J.G., Hamarneh G. (2017). BrainNetCNN: Convolutional neural networks for brain networks; towards predicting neurodevelopment. NeuroImage.

[42] He K., Zhang X., Ren S., Sun J. (2015). Deep residual learning for image recognition. arXiv.

[43] Talo M., Baloglu U.B., Yıldırım Ö., Acharya U.R. (2019). Application of deep transfer learning for automated brain abnormality classification using MR images. Cogn. Syst. Res..

[44] Huang G., Liu Z., van der Maaten L., Weinberger K.Q. (2017). Densely Connected Convolutional Networks. Proceedings of the CVPR 2017, IEEE Conference on Computer Vision and Pattern Recognition.

[45] Howard A.G., Zhu M., Chen B., Kalenichenko D., Wang W., Weyand T., Andreetto M., Adam H. (2017). Mobilenets: Efficient Convolutional Neural Networks for Mobile Vision Applications. arXiv.

[46] Li Y., Huang H., Xie Q., Yao L., Chen Q. (2018). Research on a Surface Defect Detection Algorithm Based on MobileNet-SSD. Appl. Sci..

[47] Ragab D.A., Attallah O., Sharkas M., Ren J., Marshall S. (2021). A Framework for Breast Cancer Classification Using Multi-DCNNs. Comput. Biol. Med..

[48] Attallah O., Sharkas M.A., GadElkarim H. (2019). Fetal Brain Abnormality Classification from MRI Images of Different Gestational Age. Brain Sci..

[49] Lahmiri S., Boukadoum M. (2013). Hybrid Discrete Wavelet Transform and Gabor Filter Banks Processing for Features Extraction from Biomedical Images. J. Med. Eng..

[50] Srivastava V., Purwar R.K. (2017). A Five-Level Wavelet Decomposition and Dimensional Reduction Approach for Feature Extraction and Classification of MR and CT Scan Images. Appl. Comput. Intell. Soft Comput..

[51] Thakral S., Manhas P. (2018). Image Processing by Using Different Types of Discrete Wavelet Transform. Proceedings of the Communications in Computer and Information Science.

[52] Jin Y., Angelini E., Laine A. (2006). Wavelets in Medical Image Processing: Denoising, Segmentation, and Registration. Handbook of Biomedical Image Analysis.

[53] Singh R., Khare A. (2013). Multiscale Medical Image Fusion in Wavelet Domain. Sci. World J..

[54] Chervyakov N., Lyakhov P., Kaplun D., Butusov D., Nagornov N. (2018). Analysis of the Quantization Noise in Discrete Wavelet Transform Filters for Image Processing. Electronics.

[55] Aydoğdu Ö., Ekinci M. (2020). An Approach for Streaming Data Feature Extraction Based on Discrete Cosine Transform and Particle Swarm Optimization. Symmetry.

[56] Vishwakarma V.P., Goel T. (2018). An efficient hybrid DWT-fuzzy filter in DCT domain based illumination normalization for face recognition. Multimed. Tools Appl..

[57] Zhang X., Peng F., Long M. (2018). Robust Coverless Image Steganography Based on DCT and LDA Topic Classification. IEEE Trans. Multimedia.

[58] Dabbaghchian S., Ghaemmaghami M.P., Aghagolzadeh A. (2010). Feature Extraction Using Discrete Cosine Transform and Dis-crimination Power Analysis with a Face Recognition Technology. Pattern Recognit..

[59] Attallah O., GadElkarim H., Sharkas M.A. (2018). Detecting and Classifying Fetal Brain Abnormalities Using Machine Learning Techniques. Proceedings of the 2018 17th IEEE International Conference on Machine Learning and Applications (ICMLA).

[60] Colquhoun D. (2014). An investigation of the false discovery rate and the misinterpretation of p-values. R. Soc. Open Sci..

[61] Attallah O. (2020). An Effective Mental Stress State Detection and Evaluation System Using Minimum Number of Frontal Brain Electrodes. Diagnostics.

